# Impact of preoperative frailty on new disability or death after cardiac surgery in elderly patients: a prospective cohort study

**DOI:** 10.3389/fmed.2025.1526896

**Published:** 2025-02-19

**Authors:** Wenwen Ma, Weikang Shui, Qian Peng, Chaoyang Zhu, Wenjing Zhao, Guanglei Fan, Shanshan Zhu

**Affiliations:** ^1^Department of Anaesthesiology, Xuzhou Cancer Hospital, Xuzhou, China; ^2^Department of Anesthesiology, Wuxi People’s Hospital Affiliated to Nanjing Medical University, Wuxi, China; ^3^Department of Pediatrics, Xuzhou Children’s Hospital, Xuzhou, China; ^4^Department of Intensive Care Medicine, Affiliated Hospital of Xuzhou Medical University, Xuzhou, China

**Keywords:** aging, frailty, disability, cardiac surgery, patient-centered outcome

## Abstract

**Background:**

Disability may be a potential adverse outcome of exposure to stressors in frail patients, and assessment of frailty may provide additional information for preoperative decision-making, but there is a lack of research on the impact of preoperative frailty on death or new disability after cardiac surgery. The main objective of this study was to evaluate the effect of preoperative frailty on short-term death or new disability after cardiac surgery in elderly individuals.

**Patients and methods:**

This prospective cohort study included 351 patients aged ≥60 years who were scheduled to undergo elective open heart surgery at the Affiliated Hospital of Xuzhou Medical University from March 2023 to March 2024. Patients were examined prospectively using the Comprehensive Assessment of Frailty (CAF) score, which separated patients into frail and non-frail groups. The primary outcome was 90-day disability or death. Multivariate logistic regression models were used to estimate the association between frailty and 90-day new disability or death.

**Results:**

An assessment of frailty was performed on 351 patients, and 325 patients were included in the final analysis. The prevalence of frailty was found to be 23.08%. New disability or death occurred within 90 days after surgery in 41 (12.6%) of our patients. In multivariate analysis, frailty [OR, 3.31; 95% CI, 1.43–7.62] was independently associated with 90-day new disability or death. Empirical ROC analysis showed that CAF (AUC = 0.762) predicted 90-day new disability or death postoperatively more reliably than the traditional risk assessment tools ASA + age (AUC = 0.656) and EuroSCORE II (AUC = 0.643).

**Conclusion:**

The study demonstrates that preoperative frailty, bypass time, diabetes, BMI and EuroSCORE II are independent risk factors for 90-day new disability or death after cardiac surgery in elderly patients. Notably, frailty was a more effective predictor of 90-day new disability or death than the traditional risk predictors EuroSCORE II and ASA + age.

## Introduction

Compared to other surgeries, cardiac surgery is characterized by high risk and difficulty and is a highly volatile event. Patients undergoing cardiac surgery are often exposed to strong external stressors, such as extracorporeal circulation, sternotomy, hypothermia, and prolonged anesthesia and operating time, which deal a severe blow to the body’s overall homeostasis and cause damage to vital organs. However, in recent years, with the advances in surgical techniques, anesthesia management and postoperative rehabilitation care, appropriate conditions have been created to improve the prognosis of cardiac surgery. Therefore, perioperative physicians should do an excellent job of preoperative risk assessment and take effective measures in all aspects of the perioperative period to mitigate the stress injury suffered by cardiac surgery patients. Common preoperative risk assessment tools for cardiac surgery include the European System for Cardiac Operative Risk Evaluation II (EuroSCORE II), the Society of Thoracic Surgeons score (STS), and the ASA class. Still, these tools lack physiological assessment (1, 2).

Frailty is defined as a clinical condition in older people in which physiological reserves decline, resulting in increased vulnerability, decreased resistance to stress, and impaired ability to maintain or restore homeostasis after stress (3, 4). Previous studies have shown that the prevalence of frailty in elderly patients undergoing major surgery is approximately 20–40% (5, 6) and that elderly patients with preoperative comorbidities of frailty have higher rates of postoperative complications and mortality (7). However, it is reassuring to know that frailty is not a reversible functional state like age and comorbidities and that preoperative physiologic reserve can be increased with appropriate nutritional support and rehabilitation (8).

Due to the increasing standard of living, people are no longer only concerned with whether the surgery can be successful or not but rather with good independent mobility and quality of life after the surgery, and the desire to avoid a new disability is especially urgent. Disability is defined by the difficulty in performing activities of daily living or the development of various limitations. Currently, disability is often assessed using the standard sets of Activities of Daily Living (ADLs) and Instrumental Activities of Daily Living (IADLs) (9, 10), but both lack an assessment of social participation, which is included in the World Health Organization Disability Assessment Scale, second edition (WHODAS 2.0) (11, 12). Recent prospective studies have shown that preoperative disability is associated with patient self-reported death or new disability after surgery in non-cardiac patients (13, 14). Thus, disability may be a potential adverse outcome of exposure to stressors in frail patients, and assessment of frailty may provide additional information for preoperative decision-making, but there is a lack of research on the impact of preoperative frailty on death or new disability after cardiac surgery.

The main objective of this study was to evaluate the effect of preoperative frailty on short-term death or new disability after cardiac surgery in elderly individuals, to provide some reference value for perioperative risk assessment and decision-making, and to achieve the goal of improving patients’ postoperative recovery.

## Materials and methods

### Study design and study population

The study was approved by the Ethics Committee of the Affiliated Hospital of Xuzhou Medical University (XYFY2023-KL044-01) and registered in the Chinese Clinical Trials Registry (ChiCTR2300069382). We adhered to the reporting requirements established by the Strengthening the Reporting of Observational Studies in Epidemiology (15). Written informed consent was obtained from each participant prior to enrollment. Patients were recruited between March 2023 and March 2024.

Elderly (≥60 years) patients undergoing elective open heart surgery were eligible for enrollment. The exclusion criteria were as follows: Preoperative refusal to participate in this study or communication difficulties; inability to complete a frailty assessment due to absolute bed rest; preoperative comorbidities of severe hepatic and renal dysfunction; preoperative history of IABP (Intra-Aortic-Balloon-Pump), mechanical ventilation, or pacemaker implantation; those who are unable to be reached or refuse to cooperate with postoperative telephone follow-up.

### Frailty and disability assessment

During the preoperative assessment, in addition to routine examinations, all consenting participants underwent an assessment of the CAF and the WHO Disability Assessment Scale 2.0 (WHODAS) by a researcher who was unaware of the content of the postoperative follow-up; the methodology of the CAF assessment is described in the study by Sündermann et al. (16). The CAF can be broadly viewed as a composite of three frailty assessment scales [FP (Frailty Phenotype) (17), CFS (Canadian Clinical Frailty Scale) (18), and MPPT (Modified Physical Performance Test)], consisting primarily of biomarker assessments (serum albumin level, serum creatinine level, BMI, and FEV1), physical tests of fatigue, activity level, gait speed, grip strength, and balance stability, and including measures of the CFS. The total score of the CAF was 35 points, with patients scoring ≥11 being frail and those scoring <11 being non-frail.

The baseline assessment of preoperative disability was determined using the WHO Disability Assessment Scale 2.0 (WHODAS) (11–13). The WHODAS 2.0 consists of six main domains: mobility, self-care, getting by, cognition, and social participation. The 12 items of the WHODAS are scored as described in previous studies, with each item having a numerical value: none = 0; mild = 1; moderate = 2; severe = 3; and extreme = 4. The total score ranges from 0 (no disability) to 48 (total disability or death) and is then divided by 48 and multiplied by 100 to give a disability score of 0 (no disability or death). mild = 2; severe = 3; extreme = 4. The total score ranged from 0 (no disability) to 48 (total disability or death), then divided by 48 and multiplied by 100 to convert to a percentage of the disability score. Preoperative disability was defined as a WHODAS 2.0 total score percentage greater than or equal to 25%.

### Definition of outcomes

The primary outcome was the relationship between preoperative frailty and new disability or death at 3 months postoperatively. Postoperative patient follow-up was performed by an anesthesiologist who was unaware of the results of the preoperative evaluation. Telephone follow-up and electronic medical record documentation were the primary means of determining patients’ postoperative survival status. New disability at 90 days was defined as the occurrence of a WHODAS 2.0 ≥ 25% postoperatively in patients who were not disabled preoperatively; if the patient had a preoperative comorbid disability (WHODAS ≥ 25%), an increase in the percentage of WHODAS scores by 8 at 90 days postoperatively indicated the occurrence of new disability (11–13).

Secondary outcomes included (1) 90-day disability-free survival (DFS), (2) ICU length of stay, (3) postoperative non-hospital discharge, (4) postoperative length of stay, (5) major morbidity (19, 20) (Supplementary Table S1), (6) incidence of PPCs (21), (7) 90-day readmission rate, and (8) 90-day mortality.

We conducted a *post hoc* study to compare the validity of the CAF with the commonly used FP and CFS in predicting 90-day new disability or death. We asked whether the predictive validity of the traditional and rapidly assessable preoperative frailty scales (FP and CFS) would achieve the same categorical properties as the CAF, because the preoperative assessment of the CAF is time-consuming.

### Other variables

All patients were managed according to standard cardiac surgery protocols, with pre-operative perioperative risk assessment followed by anesthesia for surgery, post-operative transfer to the surgical intensive care unit, and after stabilization of vital signs, transfer to the general surgical ward for further management and routine post-operative rehabilitation for all patients.

Baseline clinical and demographic data were collected according to the protocol, including sex, age, smoking and alcohol consumption, comorbidities (diabetes, hypertension, myocardial infarction, etc.), ASA class, EuroSCORE II, pulmonary function (FEV1, FVC, and FEV1/FVC), type of surgery, and left ventricular ejection fraction (LVEF). Operating time, Cross-clamp time, bypass time, urine volume, blood loss, total fluid intake, and total fluid output were recorded during surgery. Data on ICU stay, postoperative hospital stay, non-hospital discharge, 90-day Major Morbidity, and readmission within 90 days were collected.

### Statistical analysis

Data normality was tested by visual inspection of histograms and Shapiro–Wilk’s W test. All normally distributed and skewed continuous variables were expressed as mean (SD) or median (interquartile range [IQR]). Categorical variables were indicated as frequencies (%). Comparison of continuous variables among groups was performed with the use of the Student’s *t*-test or Mann–Whitney U-test, depending on the normality of the distribution. In contrast, the Fisher’s Exact test was used to compare categorical variables.

A Least Absolute Shrinkage and Selection Operator regression analysis was conducted with statistically significant risk factors included in the univariable study to remove non-zero characteristic components. After that, a multivariate logistic regression analysis (stepwise regression method) was used to identify the 90-day new disability or death risk variables. Internal validation was carried out using the bootstrap self-sampling technique (1,000 bootstrap samples repeatedly sampled), and the model’s discrimination was tested using the relatively adjusted C-index (concordance index). The calibration curve was drawn to evaluate the model’s consistency. In addition, the inverse probability treatment weighting (IPTW) approach was used for two groups to adjust for observed possible confounding factors. Multivariable logistic regression analyses were used to obtain the IPTW-adjusted odds ratio (OR) in the IPTW-adjusted cohort. The predictive validity was assessed using the area under the receiver operating characteristic (ROC) curve (AUC). An AUC of 0.5–0.7 implies poor prediction accuracy, whereas an AUC of 0.7–0.9 suggests high prediction performance. The DeLong test compared the AUC of different models.

*p*-value < 0.05 (two-sided) was considered statistically significant. R4.1.2 and MedCalc 20.0. Statistical software was used for analysis.

### Sample size calculation

Based on the pre-test, the incidence of death or new disability at 90 days after cardiac surgery in elderly individuals is about 10%, the multivariate regression model includes at least 5 outcome events for each variable (22), a total sample size of 250 is required (5*5/10% = 250), and considering the 15% dropout rate, 250/(1–15%) = 295 patients are proposed to be included in this study.

### Missing data

We used complete cases for the initial analysis, and preoperative baseline data were complete for all participants. A total of 351 individuals were included in the study, and final data were complete for 325 individuals. Our overall proportion of missing values was small, our analysis was based on all available data without imputation.

## Results

### Baseline characteristics

**Figure 1:**
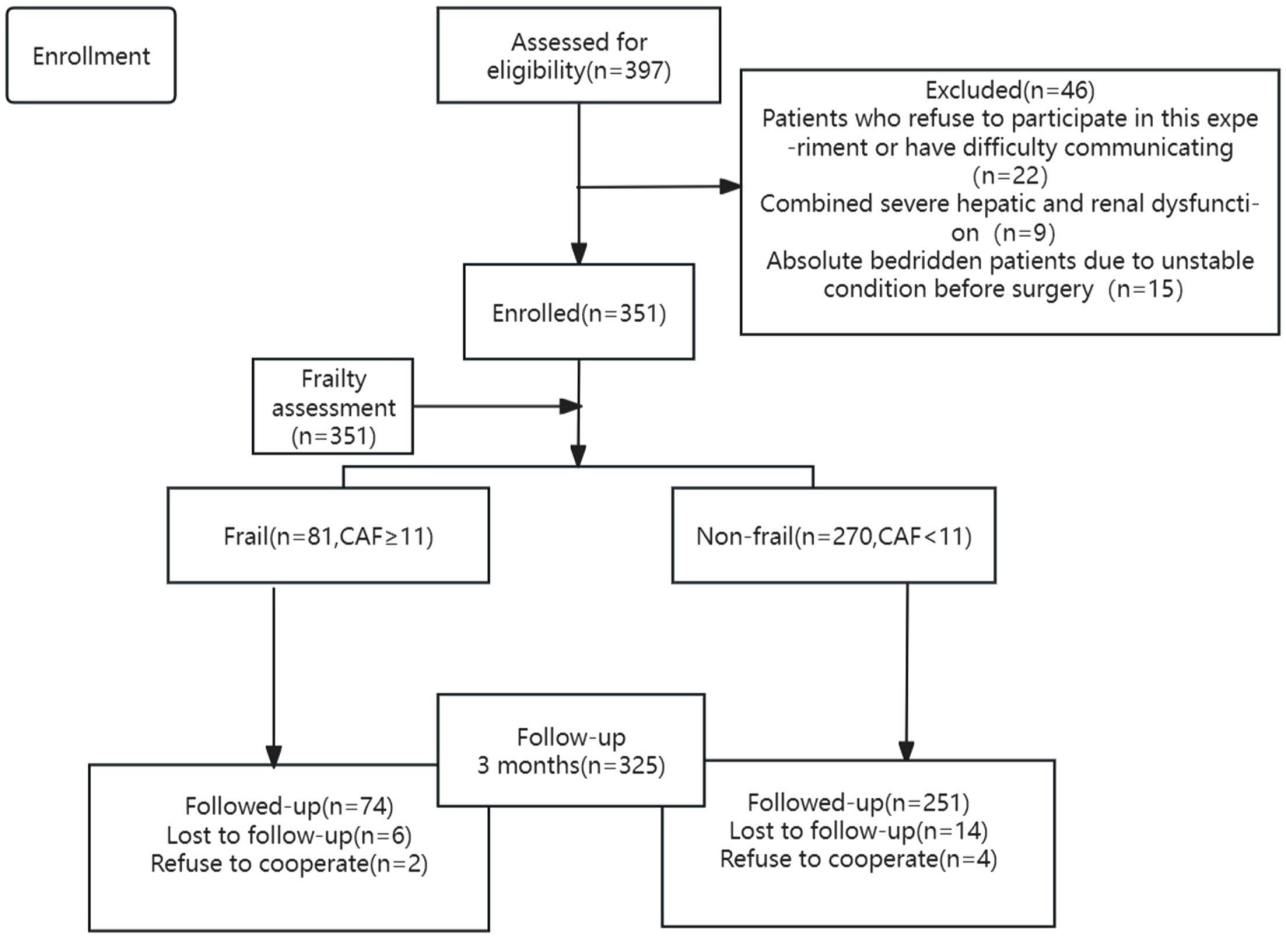
Study participant flow diagram (CAF: Comprehensive assessment of frailty score).

An assessment of frailty was performed on 351 patients, and 325 patients were included in the final analysis (Figure 1). The prevalence of frailty was found to be 23.08%. Table 1 shows that age, proportion of female, NT-ProBNP, hsTnT, stroke/TIA, myocardial infarction, diabetes, proportion of preoperative disability, NYHA class, ASA class, EuroSCORE II, and duration of surgery were higher in patients in the frail group compared with those in the non-frail group. The rates of alcohol consumption, hemoglobin, albumin, FEV1, and FVC were lower (*p* < 0.05), while all other factors were not statistically different (*p* > 0.05).

**Table 1:** Clinical characteristics of study participants.

Variables	Total (*n* = 351)	Non-frail (*n* = 270)	Frail (*n* = 81)	*p*
Age (year)	66.8 ± 5.2	66.2 ± 5.1	68.7 ± 5.1	<0.001
Sex (female)	119 (33.9)	77 (28.5)	42 (51.9)	<0.001
BMI (kg/m^2^)	24.4 ± 3.5	24.4 ± 3.4	24.6 ± 3.7	0.704
Smoke	135 (38.5)	110 (40.7)	25 (30.9)	0.109
Alcohol	100 (28.5)	85 (31.5)	15 (18.5)	0.023
LVEF (%)	58.0 ± 7.9	58.4 ± 8.0	56.6 ± 7.4	0.082
FEV1 (L)	2.3 ± 0.7	2.4 ± 0.7	1.9 ± 0.7	<0.001
FVC (L)	2.6 ± 0.9	2.7 ± 0.9	2.2 ± 0.8	<0.001
FEV_1_/FVC (%)	86.6 ± 13.6	87.1 ± 12.4	84.6 ± 17.2	0.164
NT-ProBNP (pg/mL)	742.9 ± 978.8	677.8 ± 927.6	963.3 ± 1113.8	0.023
hsTnT (ng/L)	26.5 ± 54.0	22.8 ± 51.8	38.6 ± 59.4	0.022
Hemoglobin (g/L)	134.9 ± 16.0	138.2 ± 14.4	123.8 ± 16.1	<0.001
Albumin (g/L)	41.9 ± 3.7	42.3 ± 3.7	40.5 ± 3.4	<0.001
Stroke/TIA	155 (44.2)	111 (41.1)	44 (54.3)	0.036
PAH	83 (23.6)	63 (23.3)	20 (24.7)	0.801
Bronchial disease	22 (6.3)	14 (5.2)	8 (9.9)	0.127
Myocardial infarction	49 (14.0)	31 (11.5)	18 (22.2)	0.014
Atrial fibrillation	44 (12.5)	35 (13)	9 (11.1)	0.659
Sleep apnea	36 (10.3)	24 (8.9)	12 (14.8)	0.123
Diabetes	84 (23.9)	55 (20.4)	29 (35.8)	0.004
Hypertension	184 (52.4)	138 (51.1)	46 (56.8)	0.369
Cough and sputum	73 (20.8)	51 (18.9)	22 (27.2)	0.108
CKD	21 (6.0)	14 (5.2)	7 (8.8)	0.283
Type of operation				0.925
CABG	195 (55.6)	151 (55.9)	44 (54.3)	
Valve	127 (36.2)	97 (35.9)	30 (37)	
CABG + Valve	26 (7.4)	20 (7.4)	6 (7.4)	
Other	3 (0.9)	2 (0.7)	1 (1.2)	
CAF	8.6 ± 5.4	6.2 ± 2.1	16.8 ± 5.1	<0.001
FP	1.0 (0.0, 2.0)	1.0 (0.0, 1.0)	3.0 (2.0, 4.0)	<0.001
CFS	3.0 (3.0, 4.0)	3.0 (3.0, 3.0)	4.0 (4.0, 5.0)	<0.001
EuroSCORE II (%)	1.2 (0.9, 1.7)	1.1 (0.8, 1.5)	2.0 (1.4, 2.8)	<0.001
WHODAS	5.0 (2.0, 9.0)	4.0 (1.0, 7.0)	14.0 (9.0, 20.0)	<0.001
Preoperative disability (WHODAS ≥ 25%)	70 (20.1)	17 (6.3)	53 (66.2)	<0.001
ASA class				<0.001
2	8 (2.3)	8 (3)	0 (0)	
3	286 (81.5)	243 (90)	43 (53.1)	
4	57 (16.2)	19 (7)	38 (46.9)	
NYHA ≥ 3	110 (31.5)	54 (20.1)	56 (69.1)	<0.001
Off-pump surgery	146 (41.6)	110 (40.7)	36 (44.4)	0.553
Cross-clamp time (min)	42.0 (0.0, 86.0)	42.0 (0.0, 82.0)	45.0 (0.0, 94.0)	0.794
Bypass time (min)	70.0 (0.0, 122.0)	71.0 (0.0, 119.0)	60.0 (0.0, 130.0)	0.807
Time of operation (h)	5.2 ± 1.5	5.1 ± 1.4	5.5 ± 1.5	0.044
Total liquid output (L)	2.00 (1.46, 2.345)	2.00 (1.40, 2.315)	2.02 (1.60, 2.40)	0.916
Total fluid intake (L)	3.15 (2.75, 3.909)	3.150 (2.70, 3.90)	3.20 (2.80, 3.953)	0.376
Urine output (L)	1.50 (1.00, 2.00)	1.50 (1.00, 2.00)	1.50 (1.00, 2.00)	0.695
Blood loss (L)	0.50 (0.35, 0.70)	0.485 (0.342, 0.60)	0.50 (0.40, 0.80)	0.212

Data: Means ± standard deviations, absolute rate, and percentage. Statistical significance was defined as *p* < 0.05. BMI, Body Mass Index; LVEF, Left Ventricular Ejection Fraction; FVC, Forced Vital Capacity; FEV1, Forced Expiratory Volume In 1 s; TIA, Transient Ischemic Attacks; PAH, Pulmonary Arterial Hypertension; CKD, Chronic Kidney Diseases; CABG, Coronary Artery Bypass Graft; CAF, Comprehensive Assessment of Frailty; FP, Frailty Phenotype; CFS, Clinical Frailty Scale; EuroSCORE II, European System for Cardiac Operative Risk Evaluation II; WHODAS, WHO Disability Assessment Schedule; ASA, American Society of Anesthesiologists; NYHA, New York Heart Association.

### Associations of frailty with post-operative outcomes

New disability or death occurred within 90 days after surgery in 41 (12.6%) of our patients, including 23 (33.1%) in the frail group and 18 (7.2%) in the non-frail group of patients; there was a significant difference in new disability or death at 90 days between the groups (Table 2, *p* < 0.05). Table 2 shows that patients in the frail group had higher WHODAS scores, postoperative pulmonary complications, major morbidity at 90 days, ICU stay, postoperative hospitalization, in-hospital mortality, mortality within 90 days, and incidence of readmission within 90 days (*p* < 0.05), while disability-free survival (DFS) at 90 days postoperatively was lower (*p* < 0.05).

**Table 2:** Postoperative outcomes.

Variables	Total (*n* = 325)	Non-frail (*n* = 251)	Frail (*n* = 74)	*p*
WHODAS in 90 day	3.0 (1.0, 7.0)	2.0 (0.0, 4.5)	12.0 (4.0, 33.5)	<0.001
New disability or death	41 (12.6)	18 (7.2)	23 (31.1)	<0.001
Disability-free survival (DFS)	262 (80.6)	226 (90)	36 (48.6)	<0.001
90-day major morbidity	80 (24.6)	47 (18.7)	33 (44.6)	<0.001
PPCs in 90 day	111 (34.2)	66 (26.3)	45 (60.8)	<0.001
Pulmonary infection	48 (14.8)	26 (10.4)	22 (29.7)	<0.001
Prolonged mechanical ventilation	32 (9.8)	13 (5.2)	19 (25.7)	<0.001
Deep sternal wound infection	17 (5.2)	11 (4.4)	6 (8.1)	0.234
New stroke	3 (0.9)	1 (0.4)	2 (2.7)	0.131
Delirium	42 (12.9)	22 (8.8)	20 (27)	<0.001
AKI	52 (16.0)	36 (14.3)	16 (21.6)	0.133
Sepsis	10 (3.1)	5 (2)	5 (6.8)	0.052
Re-exploration for bleeding	12 (3.7)	8 (3.2)	4 (5.4)	0.480
Postoperative hospital stay	10.0 (8.0, 12.0)	9.0 (8.0, 11.0)	11.0 (9.0, 14.0)	0.003
ICU stay	17.0 (17.0, 19.0)	17.0 (17.0, 19.0)	18.0 (17.0, 48.0)	<0.001
In hospital mortality	15 (4.6)	6 (2.4)	9 (12.2)	0.002
Death of 3 month	24 (7.4)	10 (4)	14 (18.9)	<0.001
Readmission in 90 days	40 (12.3)	24 (9.6)	16 (21.6)	0.006

Data: Means ± standard deviations, absolute rate, and percentage. Statistical significance was defined as *p* < 0.05. WHODAS, WHO Disability Assessment Schedule; PPCs, Postoperative Pulmonary Complications; AKI, Acute Kidney Injury; ICU, Intensive Care Unit.

### Risk factors associated with 90-day new disability or death

**Figure 2:**
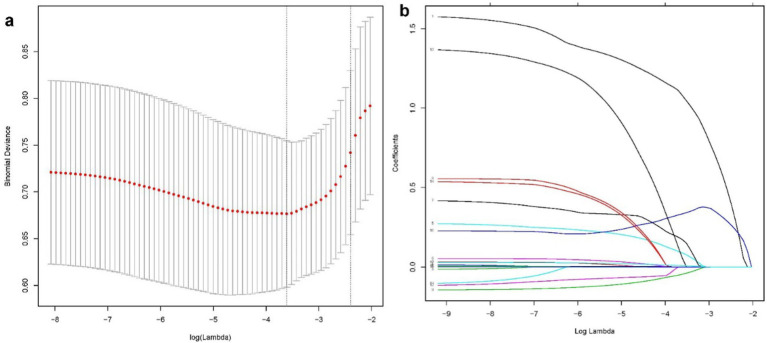
The LASSO binary logistic regression model is used to select variables. A coefficient profile plot **(A)** was used to display the log (lambda) series. Eight variables with nonzero coefficients were chosen using optimal lambda. To confirm the optimal parameter (lambda) in the LASSO model, the partial likelihood deviance (binomial deviance) curve was plotted against log (lambda), and dotted vertical lines were drawn based on a min criteria **(B)**.

**Figure 3:**
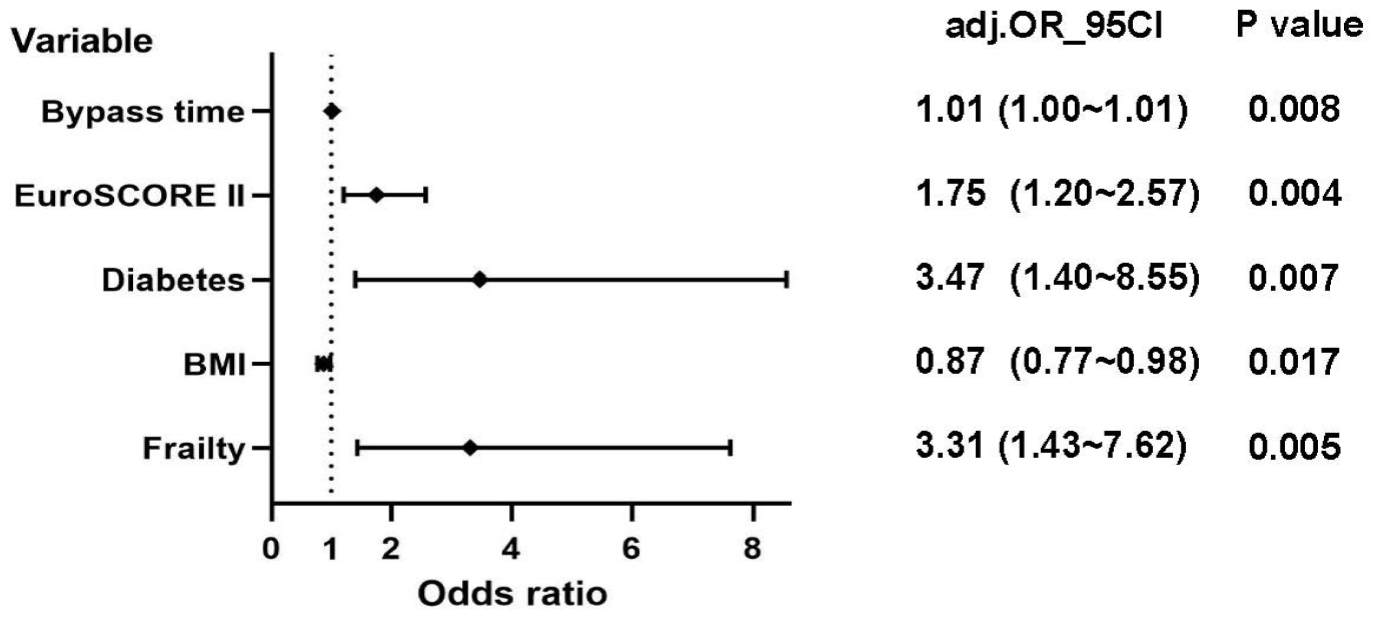
Logistic regression analysis of 90-day new disability or death (EuroSCORE II: European System for Cardiac Operative Risk Evaluation II; BMI: Body Mass Index).

According to the Least Absolute Shrinkage and Selection Operator regression analysis (Figure 2), we selected eight non-zero characteristic variables including frailty, EuroSCORE II, diabetes, Creatinine, ASA class, BMI, time of operation and bypass time (Table 3). Then, taking these eight predictors and conducting a multifactor logistic regression using a stepwise regression method approach, five meaningful variables were finally identified (Figure 2). In multivariate analysis, frailty [OR, 3.31; 95% CI, 1.43–7.62], bypass time (OR, 1.01; 95% CI, 1.00–1.01), BMI (OR, 0.87; 95% CI, 0.77–0. 98), diabetes (OR, 3.47; 95% CI, 1.40–8.55), and EuroSCORE II (OR, 1.75; 95% CI, 1.20–2.57) were independently associated with 90-day new disability or death (Figure 3). Frailty (OR, 3.15; 95% CI, 1.55–6.43) was also independently associated with 90-day new disability or death in a multivariate analysis of the inverse probability weighted adjusted cohort (Table 4).

**Table 3:** Coefficients and Lambda.min value of the LASSO regression.

Variable num	Variable name	Coefficient	lambda.type	lambda.value
1	Frailty	1.08	lambda.min	0.027
10	EuroSCORE II	0.334	lambda.min	0.027
13	Diabetes	0.055	lambda.min	0.027
17	Creatinine	0.008	lambda.min	0.027
4	Bypass time	0.001	lambda.min	0.027
5	Time of operation	0.088	lambda.min	0.027
7	ASA class	0.172	lambda.min	0.027
9	BMI	−0.046	lambda.min	0.027

EuroSCORE II, European System for Cardiac Operative Risk Evaluation II; ASA, American Society of Anesthesiologists; BMI, Body Mass Index.

**Table 4:** Associations between frailty and 90-day new disability or death in the crude analysis, multivariable analysis, and propensity-score analyses.

Analysis	Odds ratio (95%CI)
Crude analysis	5.84 (2.94 ~ 11.61)
Multivariable analysis	3.31 (1.43 ~ 7.62)
Propensity-score analysis with inverse probability weighting	3.15 (1.55 ~ 6.43)

Model validation: We reported a bias-corrected concordance statistic to verify the internal validity of our primary model utilizing calibration and discrimination with a 1,000-sample bootstrapping approach. Our calibration curve revealed that our model was well-calibrated (Supplementary Figure S1). With a C-statistic of 0.824 and an optimism-corrected C-statistic of 0.810, the discriminative ability demonstrated strong model performance in predicting 90-day new disability or death.

### Predictability of postoperative 90-day new disability or death, DFS, PPCs and 90-day major morbidity by different risk assessment tools

**Figure 4:**
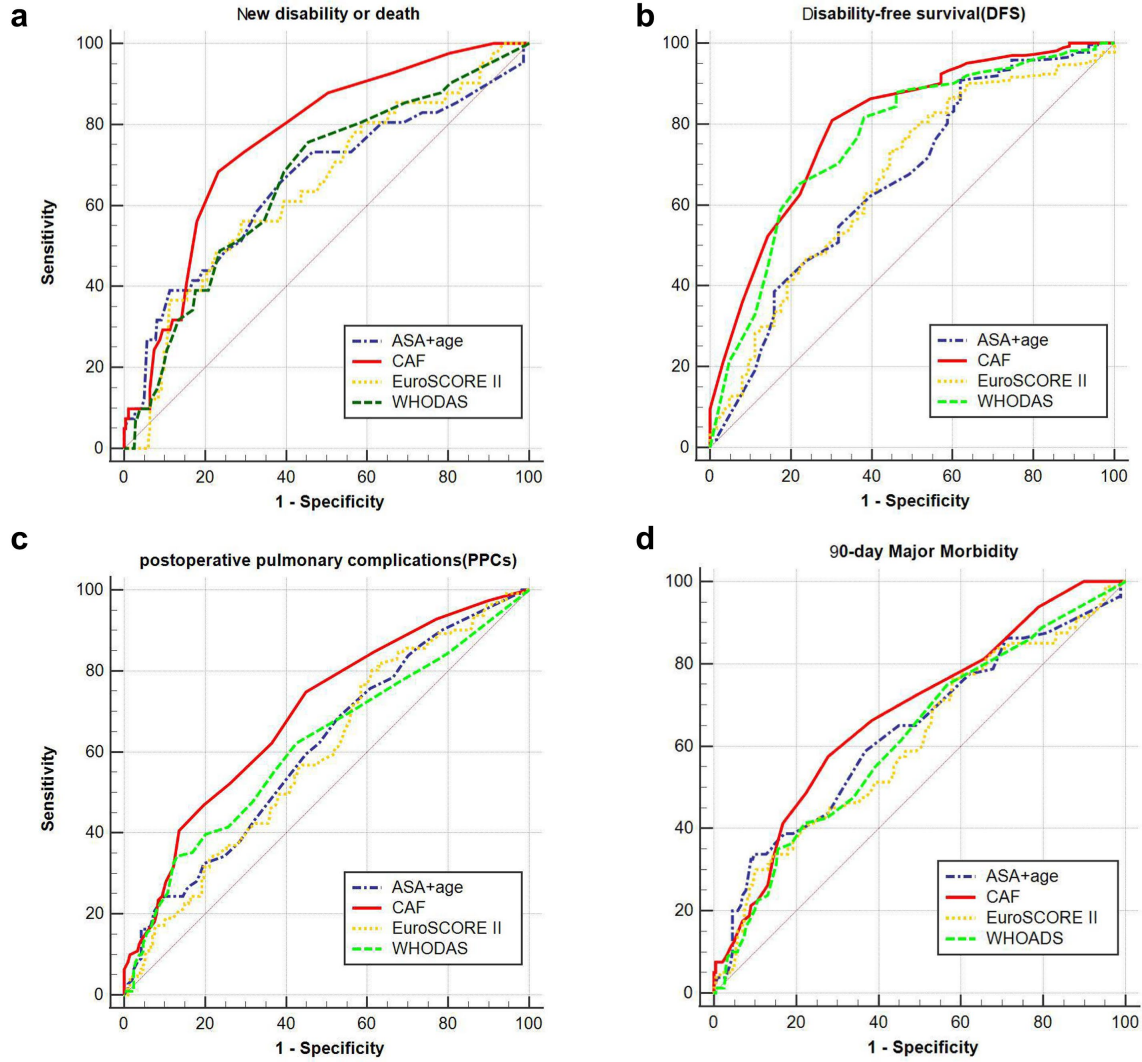
Prediction of different risk indices at different complications and 90-day new disability or death. **(A)** The area under the curve of chart-derived CAF, ASA + age, EuroSCORE II and WHODAS for DFS was 0.762 (95% CI, 0.712–0.807), 0.656 (95% CI, 0.602–0.708), 0.643 (95% CI, 0.588–0.695), and 0.662 (95% CI, 0.608–0.714), respectively. **(B)** The area under the curve of chart-derived CAF, ASA + age, EuroSCORE II and WHODAS for 90-day new disability or death was 0.799 (95% CI, 0.752–0.841), 0.668 (95% CI, 0.614–0.719), 0.668 (95% CI, 0.614–0.719), and 0.765 (95% CI, 0.715–0.810), respectively. **(C)** The area under the curve of chart-derived CAF, ASA + age, EuroSCORE II and WHODAS for PPCs was 0.694 (95% CI, 0.641–0.744), 0.608 (95% CI, 0.553–0.662), 0.594 (95% CI, 0.538–0.647), and 0.614 (95% CI, 0.559–0.668), respectively. **(D)** The area under the curve of chart-derived CAF, ASA + age, EuroSCORE II and WHODAS for 90-day Major Morbidity was 0.678 (95% CI, 0.624–0.728), 0.634 (95% CI, 0.579–0.687), 0.608 (95% CI, 0.552–0.661), and 0.619 (95% CI, 0.563–0.672), respectively (EuroSCORE II: European System for Cardiac Operative Risk Evaluation II; WHODAS WHO Disability Assessment Schedule; ASA American Society of Anesthesiologists; CAF: Comprehensive assessment of frailty score).

Empirical ROC analysis showed that CAF (AUC = 0.762) predicted 90-day new disability or death postoperatively more reliably than the traditional risk assessment tools ASA + age (AUC = 0.656), EuroSCORE II (AUC = 0.643), and WHODAS (AUC = 0. 662). The difference in the area under the curve between the first three methods and CAF was significant (*p* values of 0.039, 0.019, and 0.029, corresponding to z values of 2.064, 2.350, and 2.190, respectively). It can also be seen that the CAF was also a better predictor of DFS among the four risk assessment tools (AUC = 0.799), but was a poorer predictor of PPCs and 90-day major morbidity for all four risk assessment tools (AUC < 0.700) (Figure 4).

### *Post hoc* outcomes

Compared to the common clinical frailty assessment scales (CFS and FP), the CAF (AUC = 0.762) had superior predictive efficacy compared to CFS (AUC = 0.696) and FP (AUC = 0.686) (*p*-values of 0.036 and 0.005, corresponding to *z*-values of 2.096 and 2.819, respectively, Supplementary Figure S2).

## Discussion

The study demonstrates that preoperative frailty, bypass time, diabetes, BMI, and EuroSCORE II are independent risk factors for 90-day new disability or death after cardiac surgery in elderly patients. Notably, frailty was a more effective predictor of 90-day new disability or death than the traditional risk predictors EuroSCORE II and ASA + age.

The study indicates that frailty is an independent risk factor for new disability or death after cardiac surgery in the elderly, which is consistent with other studies. In a multicenter cohort study of 702 patients undergoing elective noncardiac surgery, frail patients had statistically significantly higher rates of new 90-day disability or death, longer hospital stays, and need for institutional discharge compared to other patient groups (13). However, this study’s surgical procedures did not include cardiac surgery. Therefore, the results cannot be applied to patients undergoing cardiac surgery. Another single-center cohort study (6) of 146 adult patients undergoing elective open-heart surgery also found that preoperative frailty significantly reduced patients’ disability-free survival at 90 days postoperatively, leading to a decrease in postoperative functional recovery and quality of life. This study, while insightful about the relationship between preoperative frailty and disability-free survival at 90 days postoperatively in patients undergoing cardiac surgery, did not have sufficient efficacy (the sample size was only 145) to explore whether frailty was an independent risk factor for new disability or death at 90 days postoperatively and to rule out the effect of preoperative patients’ comorbid disability on their postoperative functional status. The mechanisms behind frailty and postoperative self-reported disability in elderly cardiac surgery patients are unclear. These issues may be related to chronic inflammation, aging of the immune system, and endocrine dysregulation (23–25).

This study showed that the prevalence of preoperative frailty in elderly patients was 23.08%, which is consistent with previous reports in the literature (6, 16). The clinical significance of assessing preoperative frailty is to assist the perioperative physician in recognizing the patient’s preoperative risk level and making rational clinical decisions. Overall, in order to improve the prognosis of frail patients, multidisciplinary collaboration is more important (26). In frail patients at higher risk for poor functional outcomes, the cardiac surgeon decides whether minimally invasive treatment is possible (27, 28), the anesthesiologist enhances intraoperative monitoring of the patient’s vital organs (e.g., enhanced cerebral oximetry monitoring, lung-protective ventilation, and ultrasound-guided goal-directed fluid therapy) (29) and the nursing team initiates an early tailored Customized functional recovery programs (intensive pulmonary physiotherapy, early ambulation, resistance training, and nutritional support) should be initiated early by the nursing team (30). If frail patients are more stable preoperatively, we believe it is also essential to delay surgery appropriately, and studies have shown that preoperative functional rehabilitation led by the rehabilitation department can increase the patient’s physiologic reserve, thereby increasing stress resistance (30). The study also discovered that conventional risk assessment metrics, such as EuroSCORE II and ASA + age, were inadequate predictors of patient-centered outcomes, such as new disability or death. The ROC analysis clearly showed that frailty, as defined by CAF, is a more reliable predictor of adverse postoperative outcomes in elderly cardiac patients than EuroSCORE and ASA + age. Therefore, adding frailty measures, particularly CAF, to the traditional perioperative risk scoring system may improve the ability of perioperative physicians to predict relevant clinical outcomes in patients.

The present study also demonstrated that bypass time, BMI, diabetes, and EuroSCORE II were independent risk factors for 90 days after cardiac surgery in elderly patients. Longer bypass time are known to be more fatal for elderly and frail cardiac surgery patients. Previous studies (31) have shown that prolonged bypass time reflects the complexity of the surgical maneuver and may exacerbate damage to vital organs, with adverse prognostic consequences for elderly patients. In this study, only 4.9% of elderly patients were obese (BMI ≥ 30 kg/m^2^). On this basis, we found that higher BMI was associated with lower disability or death. Previous studies (32) have shown that lower body weight and obesity are associated with poor prognosis after cardiac surgery (U-shaped relationship between BMI and all-cause mortality). That said, in the present study, there were fewer obese patients whose positive association with poor outcome was masked, thus showing overall that a slightly higher BMI may contribute to survival. Diabetes is characterized by long-term insulin resistance, compensatory hyperinsulinemia, and varying degrees of hyperglycemia (33). Previous studies (34) have confirmed that diabetes is associated with patient prognosis. Patients with hyperglycemia are at increased risk for surgical site infection, pneumonia, delirium, and mortality.

There are some limitations to our study. First, the present study was a single-center, small-sample observational study with only a short-term postoperative follow-up, and a large multicenter sample is needed in the future to validate the conclusions and explore the relationship between frailty and long-term postoperative disability trajectory. Second, the CAF involves several aspects and may be time-consuming to assess preoperatively, but the *post hoc* analysis of the present study demonstrated that the CAF, although time-consuming, is a better predictor of postoperative outcomes than conventional frailty assessment scales (FP and CFS), so the CAF is recommended for the very high-risk elderly cardiac surgery population. Third, this study excluded patients who were completely bedridden preoperatively. Fourth, this study’s predictors and outcome indicators were human-rated scales, both of which are somewhat subjective. In addition, due to the nature of observational studies, there may be confounding factors that cannot be assessed.

## Conclusion

In conclusion, we found that preoperative frailty, prolonged bypass time, diabetes, BMI, and EuroSCORE II were independent risk factors for 90-day new disability or death after cardiac surgery in elderly patients. Frailty was more effective in predicting 90-day new disability or death than the traditional risk predictors EuroSCORE II and ASA + age. Preoperative assessment of frailty can assist the perioperative team in preoperative clinical decision making and provide medical support throughout the course of an elderly frail patient scheduled for cardiac surgery.

## Data Availability

The raw data supporting the conclusions of this article will be made available by the authors, without undue reservation.
