# Supplementary material for: Impact of preoperative frailty on new disability or death after cardiac surgery in elderly patients: a prospective cohort study
Source: Front Med (Lausanne). 2025 Feb 19;12:1526896. doi: 10.3389/fmed.2025.1526896 (PMC11907955; doi:10.3389/fmed.2025.1526896)
Supplement: Supplementary file 1 [file Data_Sheet_1.docx]

**Supplementary material**

**Impact of preoperative frailty on new disability or death after cardiac surgery in elderly patients: a prospective cohort study**

**Table 1.** Major morbidity definition

**Stroke:** confirmed by CT brain results;
**AKI:** new requirement for dialysis or increase in serum creatinine >153 mol/l [>2mg/dl] and >2-fold the pre-operative level)

**Deep sternal wound infection:** requirement for operative intervention and antibiotic therapy, with positive culture;

**Sepsis:** infection + SOFA ≥ 2;

**Prolonged ventilation:**invasive mechanical ventilation for >24 hours;

**Pulmonary infection:** new pulmonary infiltrate on chest x-ray plus at least two of the following: temperature > 38°5C or < 35.5°C, leukocytosis or leukopenia (white blood cells > 12,000 cells/mm3 or < 4000 cells/mm3), purulent secretions and antibiotic treatment;

**Re-exploration for bleeding:** a secondary thoracotomy was performed to control bleeding due to increased postoperative drainage.

**Delirium:** disturbed state of consciousness and cognitive dysfunction with or without agitation.

AKI,acute kidney injury;SOFA,sequential organ failure assessment.

**
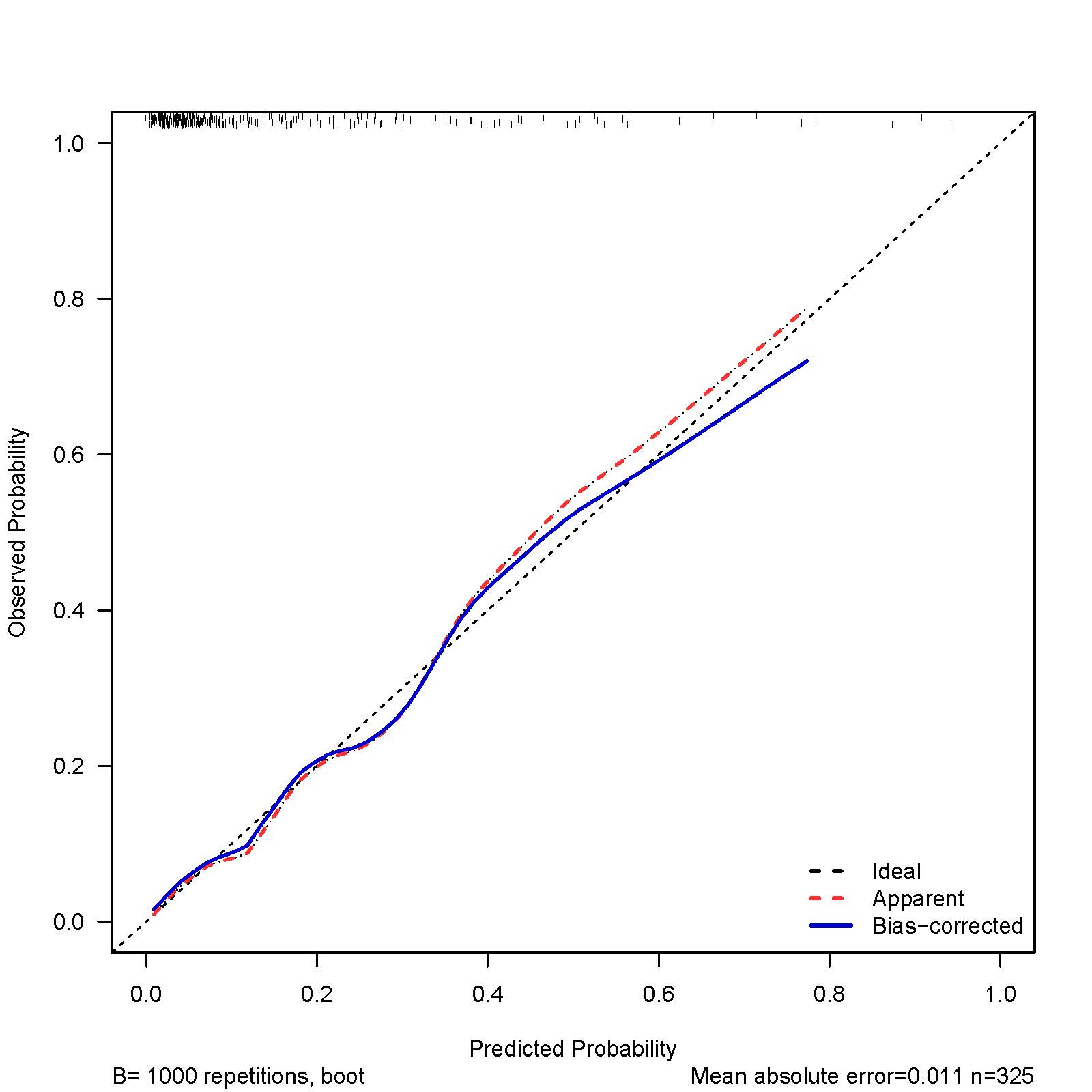
**

**Fig 1** The frequency distribution of the projected probability is represented by the x-axis. The observed probability is shown by the y-axis. In-sample calibration is shown by the "apparent" line. Where is equal to the observed probability, the "ideal" line reflects perfect prediction. The calibration after optimism correction by bootstrapping*1000 samples is represented by the "bias-corrected" line.

**
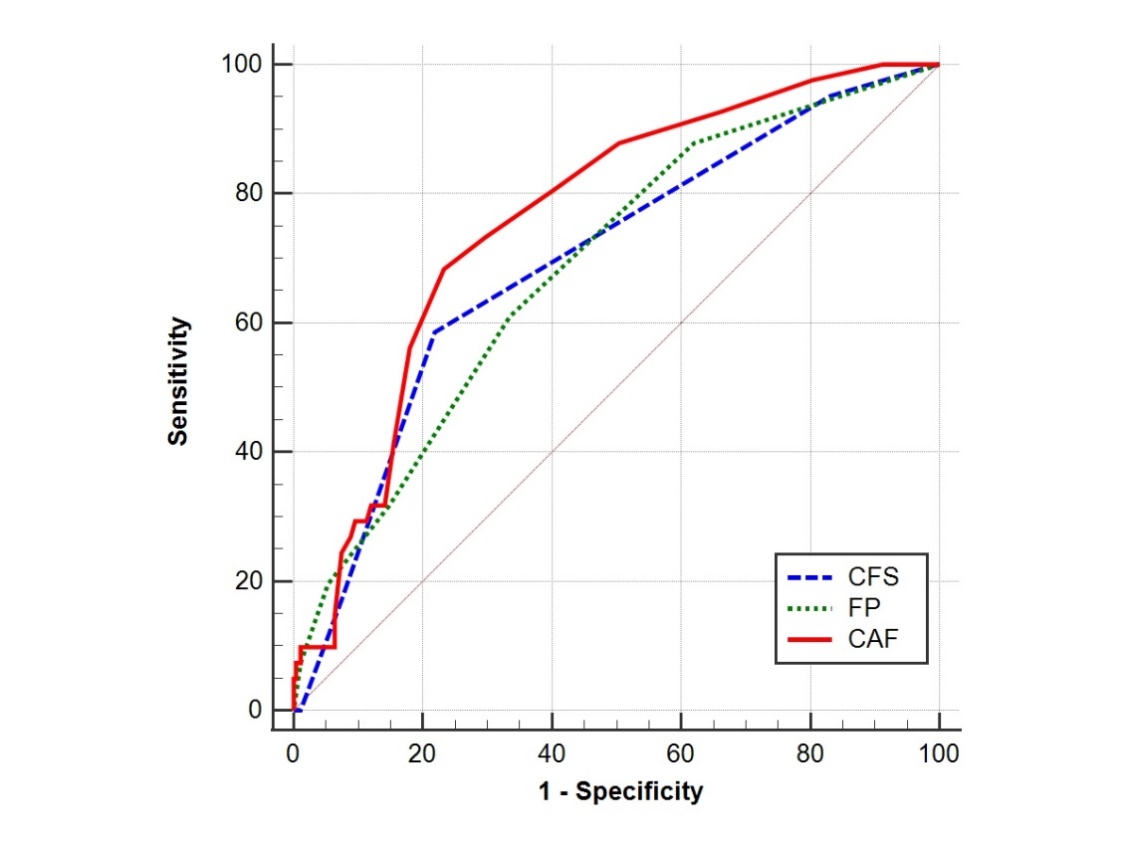
**

**Fig 2** The area under the curve of chart-derived FP,CFS and CAF for 90-day new disability or death(CAF comprehensive assessment of frailty; CFS Clinical Frailty Scale; FP Frailty Phenotype).
